# Infection with Tomato Mosaic Virus in *Nicotiana tabacum* cv. Samsun—Physiological and Molecular Approach During Early Stage of Infection

**DOI:** 10.3390/ijms27177520

**Published:** 2026-08-22

**Authors:** Wojciech Makowski, Ingrida Mažeikienė, Łucja Kmita, Edvinas Misiukevičius, Damian Adamus, Barbara Tokarz, Marta Stafiniak, Barbara Nowak, Zbigniew Gajewski, Krzysztof M. Tokarz

**Affiliations:** 1 Department of Botany, Physiology and Plant Protection, Faculty of Biotechnology and Horticulture, University of Agriculture in Krakow, al. Mickiewicza 21, 31-120 Kraków, Poland; lucja.kmita@gmail.com (Ł.K.); damian.adamus@urk.edu.pl (D.A.); barbara.tokarz@urk.edu.pl (B.T.); barbara.nowak@urk.edu.pl (B.N.); zbigniew.gajewski@urk.edu.pl (Z.G.); km.tokarz.ipbb@gmail.com (K.M.T.); 2 Department of Orchard Plant Genetics and Biotechnology, Institute of Horticulture, Lithuanian Research Centre for Agriculture and Forestry, Kaunas Str. 30, 54333 Babtai, Lithuania; ingrida.mazeikiene@lammc.lt (I.M.); edvinas.misiukevicius@lammc.lt (E.M.); 3 Department of Pharmaceutical Biology and Biotechnology, Faculty of Pharmacy, Wroclaw Medical University, Borowska 211A, 50-556 Wroclaw, Poland; marta.stafiniak@umw.edu.pl

**Keywords:** *Tobamovirus tomatotessellati*, ToMV, cultivated tobacco, photosynthetic apparatus, antioxidative enzymes, non-enzymatic antioxidants, gene expression

## Abstract

Although tomato mosaic virus (ToMV) is an economically important tobamovirus, the physiological and molecular events occurring during the asymptomatic phase of infection remain poorly understood. In this study, we investigated the early responses of *Nicotiana tabacum* cv. Samsun to ToMV infection using an integrated physiological, biochemical, photosynthetic, and molecular approach. Viral accumulation was quantified via RT-PCR. Oxidative stress markers, antioxidant systems, photosynthetic performance, and gene expression were analyzed at 7 days post inoculation (DPI), before visible symptoms developed. Although infected plants remained symptomless, ToMV was detected in 80% of inoculated plants. Early infection induced oxidative stress, evidenced by increased malondialdehyde content, reduced free amino acid levels, and enhanced activities of superoxide dismutase and peroxidase. Total glutathione, phenolic compounds, and phenylpropanoids remained unchanged, whereas flavonoid content decreased significantly. ToMV infection also impaired the photosynthetic apparatus, resulting in reduced chlorophyll and carotenoid contents, decreased electron transport efficiency, and increased energy dissipation within photosystem II. Gene expression analysis revealed significant upregulation of defense- and stress-related genes (*WRKY1*, *HSP70*, *GR*, and *DHAR*), as well as chloroplast-associated genes (*psaA* and *rbcL*). Correlation analyses demonstrated coordinated relationships among viral accumulation, oxidative stress, antioxidant responses, and photosynthetic performance. These findings provide new insights into the asymptomatic phase of ToMV infection and identify potential early markers of host responses to viral infection.

## 1. Introduction

Plant viruses represent a major constraint on global agricultural productivity, causing significant economic losses and posing a persistent challenge to crop protection strategies. Among these pathogens, members of the genus *Tobamovirus* have attracted considerable attention because of their exceptional stability, efficient mechanical transmission, and broad host range [1]. Tomato mosaic virus (*Tobamovirus tomatotessellati*, ToMV) infects economically important solanaceous crops, including *Nicotiana tabacum*, and serves as a model system for investigating plant–virus interactions at physiological, biochemical, and molecular levels [2]. Despite extensive research on ToMV biology and host responses, the early asymptomatic phase of infection, which precedes visible symptom development, remains insufficiently characterized.

A defining feature of compatible plant–virus interactions is the existence of a latent phase, during which viral replication and systemic movement occur prior to the appearance of visible symptoms. During this stage, viruses exploit host cellular machinery for replication and spread through plasmodesmata and vascular tissues [3]. Increasing evidence indicates that viral accumulation during early infection correlates with subsequent symptom severity and disease progression [4]. Importantly, asymptomatic infected plants may serve as reservoirs of inoculum, thereby facilitating virus transmission, particularly under greenhouse conditions [3]. Therefore, understanding host responses during early infection stages is critical for the development of effective diagnostic and disease management strategies.

One of the earliest plant responses to viral infection is the rapid production of reactive oxygen species (ROS), which function both as signaling molecules and as mediators of cellular damage. The resulting oxidative burst contributes to defense signaling but may also disrupt cellular homeostasis when ROS production exceeds the antioxidant capacity of the cell [5]. Excessive ROS accumulation leads to oxidative stress, resulting in lipid peroxidation, protein oxidation, and impairment of cellular structures. Malondialdehyde (MDA), a by-product of lipid peroxidation, is widely used as a biomarker of oxidative damage in plant tissues [6]. In viral infections, ROS production is often associated with disruptions in chloroplast and mitochondrial electron transport chains, which serve as major intracellular sources of reactive oxygen species [7].

To counteract oxidative stress, plants activate enzymatic antioxidant systems, including superoxide dismutase (SOD), catalase (CAT), and peroxidases (PODs), which collectively maintain cellular redox homeostasis [6]. Viral infections frequently induce changes in the activity of these enzymes, although the magnitude and coordination of the response depend on both the host–virus combination and the stage of infection [8,9,10]. Importantly, antioxidant regulation must preserve the signaling function of ROS while preventing excessive oxidative damage [9].

In addition to enzymatic defenses, non-enzymatic antioxidants, particularly glutathione and phenolic compounds, contribute substantially to cellular redox homeostasis and stress adaptation [11,12]. Their accumulation reflects the balance between defense activation, metabolic costs, and the viral exploitation of host resources. However, the coordinated contribution of enzymatic and non-enzymatic antioxidant systems during the asymptomatic phase of viral infection remains poorly understood [11].

Viral infection is accompanied by extensive metabolic reprogramming that redirects host resources towards viral replication and defense responses [13]. Alterations in amino acid pools, protein synthesis, and secondary metabolite production reflect this metabolic shift and can influence both viral replication efficiency and host resistance [14]. Understanding these metabolic adjustments is essential for elucidating the mechanisms underlying plant susceptibility or tolerance to viral infection.

A critical target of viral infection is the photosynthetic apparatus, particularly chloroplasts. Increasing evidence highlights the role of chloroplasts as key players in plant–virus interactions, acting both as sites of viral replication and as sources of defense signals [15]. Viral infection frequently leads to the degradation of photosynthetic pigments, disruption of photosystem II (PSII) function, and impairment of electron transport chains [16]. These changes result in reduced photosynthetic efficiency and contribute to symptom development, such as chlorosis and mosaic patterns.

Early viral infection also triggers extensive transcriptional reprogramming involving defense-related genes, antioxidant enzymes, and stress-responsive transcription factors [17]. WRKY transcription factors, for instance, play key roles in regulating plant immunity and coordinating responses to biotic stress. Heat shock proteins (HSPs) contribute to protein folding and stabilization under stress conditions, while genes involved in redox homeostasis, such as glutathione reductase (GR) and dehydroascorbate reductase (DHAR), are essential for maintaining cellular balance during oxidative stress [18].

Despite substantial progress in understanding individual aspects of plant–virus interactions, there remains a need for integrative studies that combine physiological, biochemical, and molecular approaches to provide a comprehensive view of early infection processes. The present study addresses this issue by investigating the early stage of ToMV infection in *Nicotiana tabacum* cv. Samsun (nn, the genotype sensitive to infection with ToMV) using a multi-level approach. By combining the quantitative analysis of viral load, the assessment of oxidative stress markers, the evaluation of enzymatic and non-enzymatic antioxidant systems, the characterization of photosynthetic performance, and gene expression profiling, this work aims to elucidate the coordinated responses of plants during the asymptomatic phase of infection. Furthermore, correlation analysis is employed to uncover functional relationships among physiological and molecular parameters, providing insights into the systemic organization of plant defense responses. This integrative approach enables a deeper understanding of the mechanisms underlying early plant responses to viral infection and contributes to the identification of potential biomarkers for early detection and resistance-breeding strategies.

## 2. Results

### 2.1. Symptoms of Infection with ToMV and Accumulation of Virus Particles

**Figure 1:**
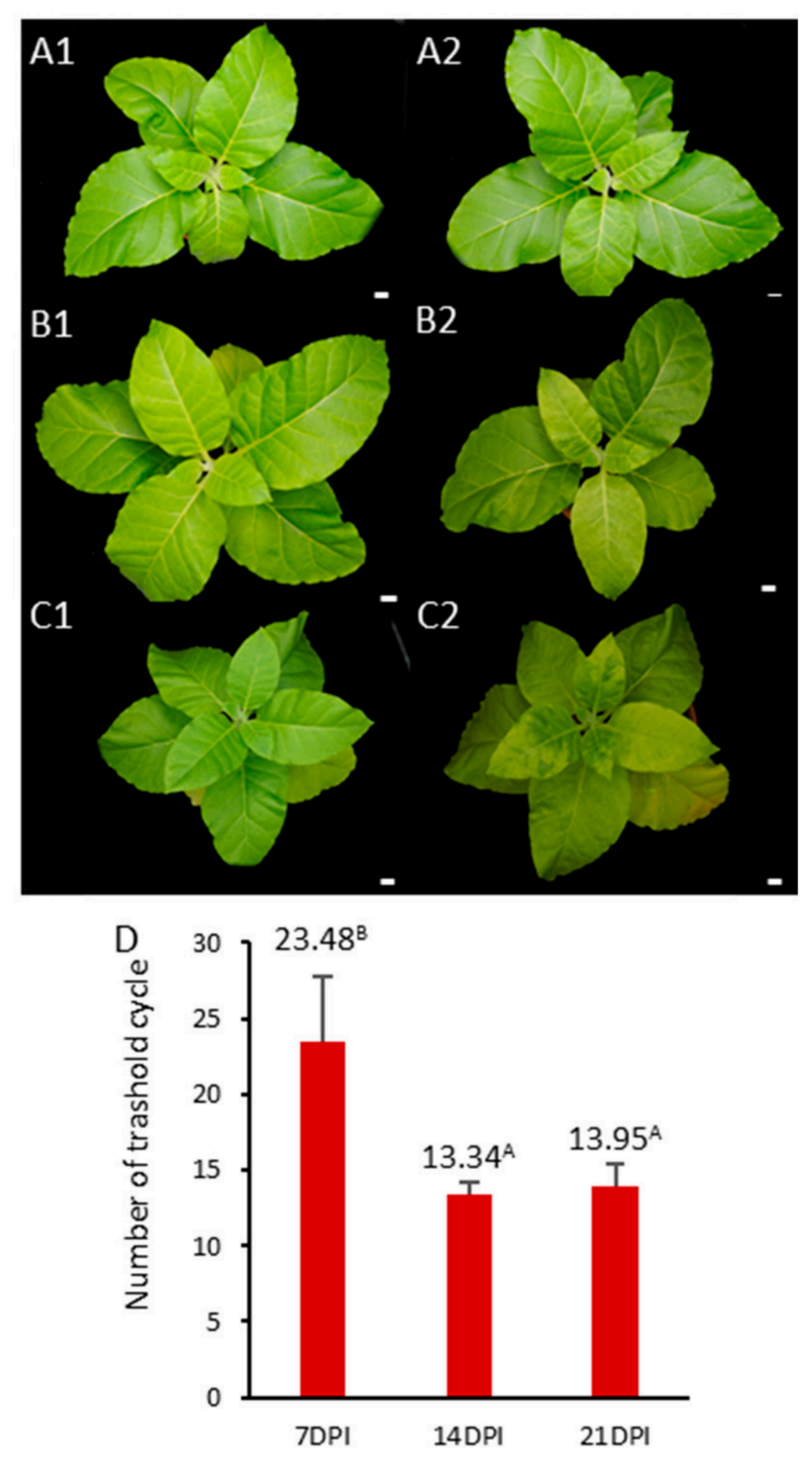
Control (mock-inoculated) *N. tabacum* plants at different timepoints of the experiment: 7 DPI (**A1**), 14 DPI (**B1**), and 21 DPI (**C1**). Virus-inoculated plants at different timepoints of the experiment: 7 DPI (**A2**), 14 DPI (**B2**) and 21 DPI (**C2**). Scale-bar = 1 cm. (**D**) Average threshold cycle numbers (Ct) at three DPI timepoints; the whiskers represent the standard errors; different letters denote significant differences according to ANOVA (*p* < 0.05).

The early detection of virus infection in plants is critical to reduce transmission, especially in indoor growing plantations. Seven days post inoculation (7 DPI), tobacco plants showed no morphological differences or symptoms of infection compared to control plants (mock-inoculated plants, MPI) at the same developmental stage (Figure 1(A1,A2)). After 9 DPI, the first systemic symptoms began to appear on the youngest leaves, which consisted of chlorotic lesions that developed into a mosaic pattern. Fully developed chlorotic mosaic was visible at 14 DPI (Figure 1(B2)), while full symptoms were observed at 21 DPI. At this stage, in addition to mosaic mottle, leaf distortion appeared (Figure 1(C2)). No disease symptoms were observed on the control (mock-inoculated) plants at 14 and 21 DPI (Figure 1(B1,C1)).

RT-PCR analyses showed the presence of viral RNA in 80% of the plants considered infected at 7 DPI (probably due to low viral RNA content or a complete lack of detection by RT-PCR). Moreover, 100% of the ToMV-infected tobacco plants were detected at 14 as well as at 21 DPI. When assessing virus replication and envelope protein gene expression in infected plants, the average Ct at 7 DPI was significantly higher than at 14 and 21 DPI (Figure 1D). The calculated errors in the variants showed that the spread of virus infection in plant leaf tissues at 7 DPI was still uneven. The virus spread stabilized at 14 DPI and remained at the same level after 21 DPI (Figure 1D).

**Figure 2:**
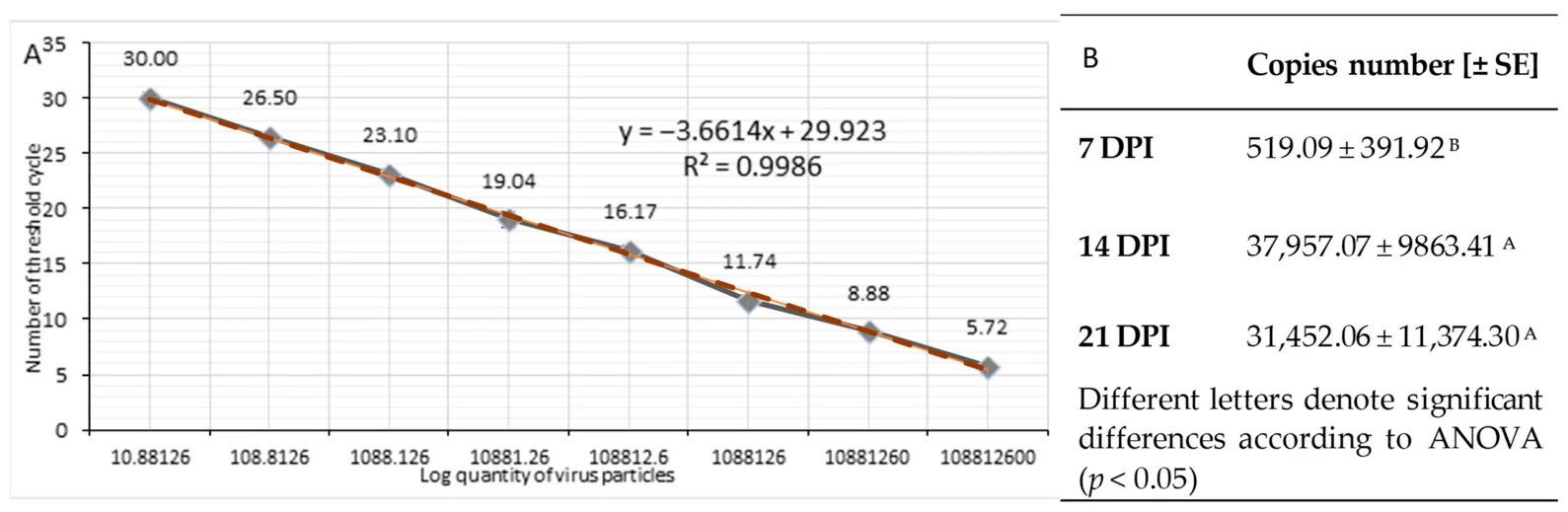
(**A**) Calibration curve of ToMV to establish viral load conversion equations. Linear regression of RT-PCR quantification cycles (dot line) and genes at known concentrations of ToMV particles (gray line). (**B**) Average number of copies, slope, and percent efficiency at three post inoculation timepoints; DPI—day post inoculation; SE—standard error.

A calibration curve generated based on the known number of virions using seven analytes allowed for a high-fidelity linear correlation, R^2^ = 0.9986, with a slope of −3.661 (−3.32 is ideal) and accurate estimation of the amount of virus particles in the plants (Figure 2A). The 88% efficiency calculated from the slope is slightly less than ideal, but completely adequate for the quantitative analysis of the virus in our case. In the initial stage of infection, the number of virions in leaves ranged from 3 to 1660. The ToMV infection peaked at 14 DPI, and the virion particles varied from 19,927 to 75,021. At 21 DPI, the variation became larger, from 6550 to 67,254 virions in the plant material, although we did not detect a significant difference between the mean values (Figure 2B).

### 2.2. Oxidative Stress Level, Protein and Free Amino Acid Concentrations, and Activity of Enzymatic Antioxidants

**Figure 3:**
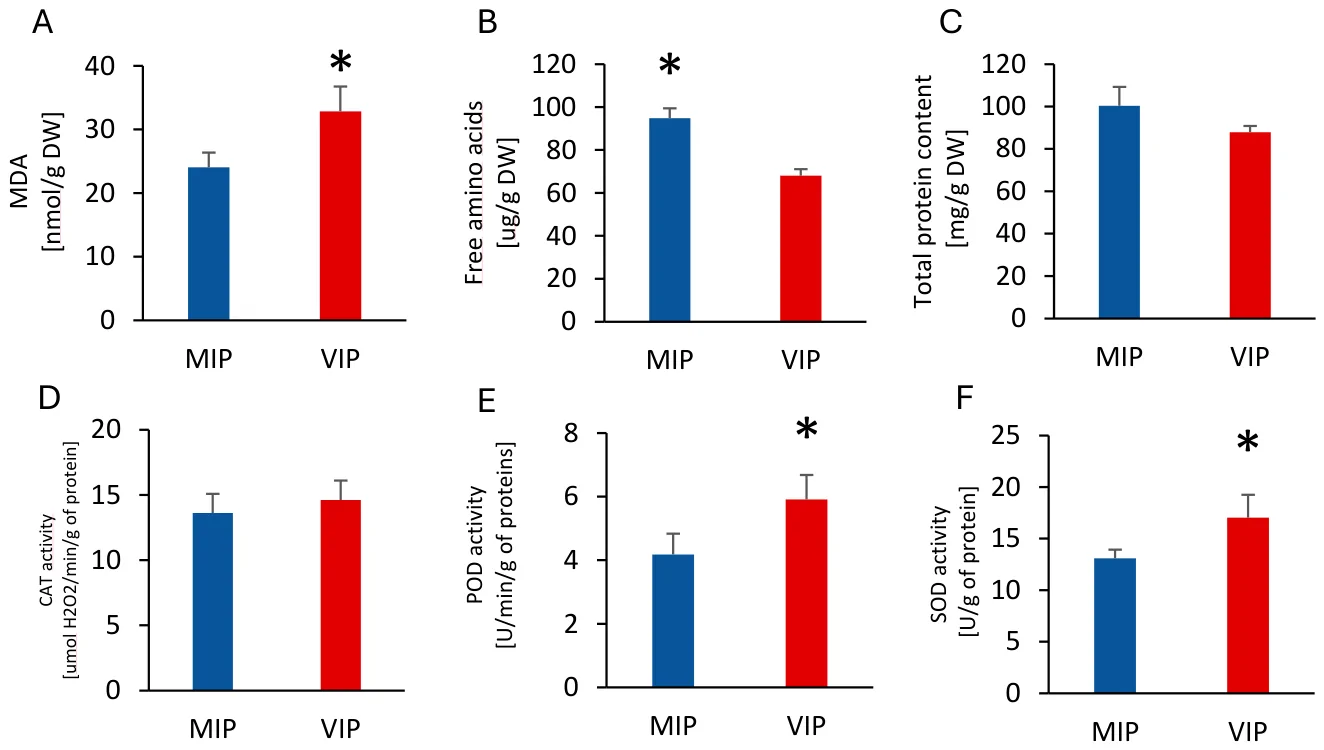
Contents of malondialdehyde (MDA) [nmol/g DW] (**A**), free amino acids [μg/g DW] (**B**), and total proteins [mg/g DW] (**C**), and activity of catalase (CAT) [μmol H_2_O_2_/min/g of protein] (**D**), peroxidase (POD) [U/min/g of protein] (**E**), and superoxide dismutase (SOD) [U/g of protein] (**F**) in mock-inoculated plants (MIPs) and virus-inoculated plants (VIPs). The asterisk indicates statistical significance of means acc. Student’s *t*-Test at *p* < 0.05; the whiskers represent the standard deviations; DW—dry weight.

According to the virus spread data, 7 DPI was a critical timepoint in the plant’s response to virus infection. Therefore, tobacco plants at this stage of infection were examined for their response in terms of stress-related parameters. The malondialdehyde content in plant tissues was found to significantly increase in ToMV-infected plants (Figure 3A). Infection also led to a decrease in free amino acid content compared to control plants (Figure 3B), while the total protein content remained unchanged (Figure 3C). In response to virus infection, the activity of two antioxidant enzymes, peroxidase and superoxide dismutase, increased early in the infection (Figure 3E,F).

### 2.3. Non-Enzymatic Antioxidant Concentrations

To characterize the physiological response of tobacco plants to ToMV infection, we analyzed the contents of non-enzymatic antioxidants. The total glutathione pool in ToMV-infected plants remained unchanged compared to control plants, and the total contents of phenolic compounds and phenylpropanoids also remained unchanged. However, infection led to reduced flavonoid accumulation in the tissues (Table 1).

### 2.4. Photosynthetic Apparatus Conditions

**Figure 4:**
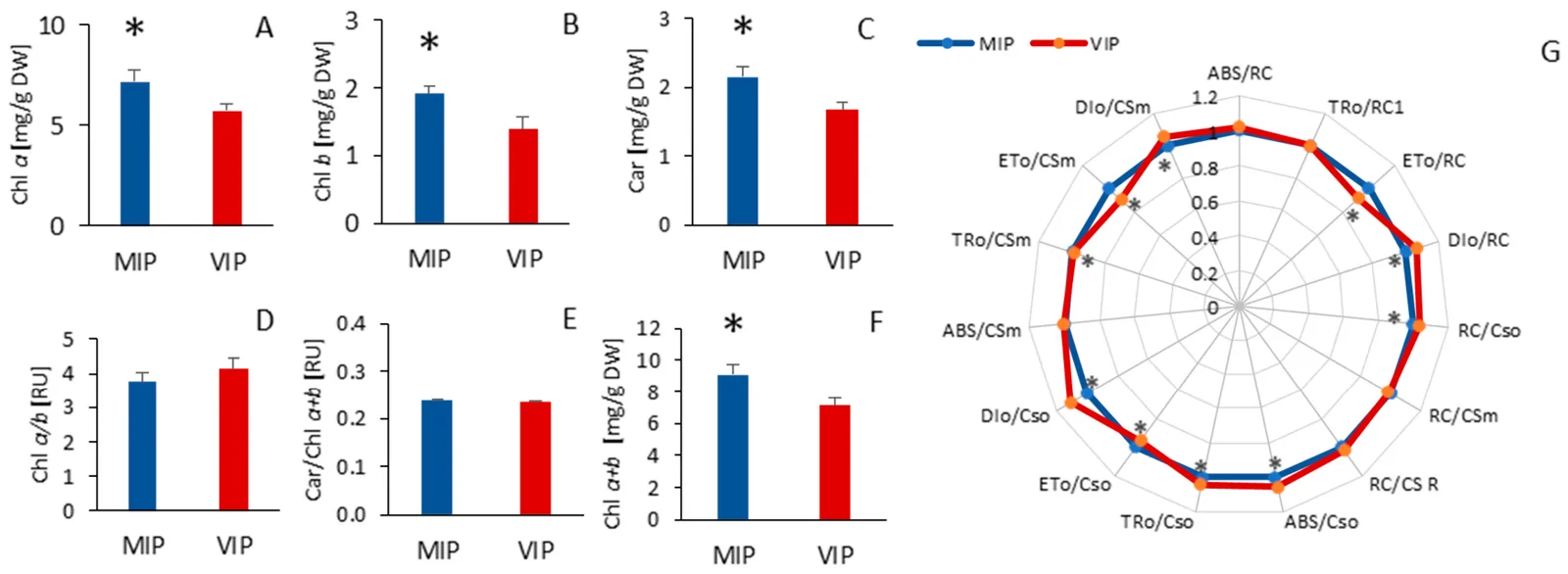
Reaction of the *Nicotiana tabacum* ‘Samsun’ photosynthetic apparatus to ToMV infection: (**A**) chlorophyll *a* content, (**B**) chlorophyll *b* content, (**C**) carotenoid content, (**D**) chlorophyll *a* to *b* ratio, (**E**) carotenoids to chlorophyll ratio [relative units], and (**F**) sum of chlorophylls [mg/g DW]. (**G**) Structural and functional parameters of PSII (all the values are expressed relative to the control—set as 1). MIP—mock-inoculated plants; VIP—virus-inoculated plants. The asterisk indicates statistical significance of means acc. Student’s *t*-Test at *p* < 0.05; the whiskers represent the standard deviation; DW—dry weight; RU—relative units.

The analysis of photosynthetic pigment contents showed a significant decrease in chlorophyll *a* (chl *a*), chlorophyll *b* (chl *b*), total chlorophyll, and carotenoids (car) in the leaves of virus-inoculated plants (Figure 4A–C,F). Meanwhile, no statistically significant differences were observed in the ratio of chlorophyll *a* to chlorophyll *b* or carotenoids to total chlorophyll (Figure 4D,E).

Chl *a* fluorescence was measured to determine the photochemical efficiency of PSII under ToMV infection. The values of the measured and calculated PSII parameters were normalized relative to the control (set as 1) and are presented in a radar chart (Figure 4G). In the presented experiment, no significant changes were observed in the specific flux of absorption (ABS/RC) and trapping flux (TRo/RC) in virus-inoculated plants compared to mock-inoculated plants (control). However, the electron transport flux (ETo/RC) decreased and the dissipated energy flux per RC (DIo/RC) increased in these plants. Furthermore, the density of active PSII reaction centers (RC/CS) increased when approximated by Fo but did not change when approximated by FM. Similarly, the absorption of energy per excited cross-section (ABS/CS) increased (approximated by Fo) and did not change (approximated by FM). The excitation energy flux trapped by PSII of a photosynthesizing sample cross-section (CS) approximated by Fo (TRo/CSo) increased in VIP leaves compared to MIP ones, but TRo/CSm decreased significantly. In turn, the electron flux transported by PSII of a photosynthesizing sample cross-section (ETo/CS) (approximated by both Fo and FM) decreased and the heat dissipation of excitation energy by PSII of a photosynthesizing sample cross-section (DIo/CS) (approximated by both Fo and FM) increased in virus-inoculated leaves (Figure 4G).

### 2.5. Differential Correlation Patterns Among Biochemical Parameters Under Viral Infection

**Figure 5:**
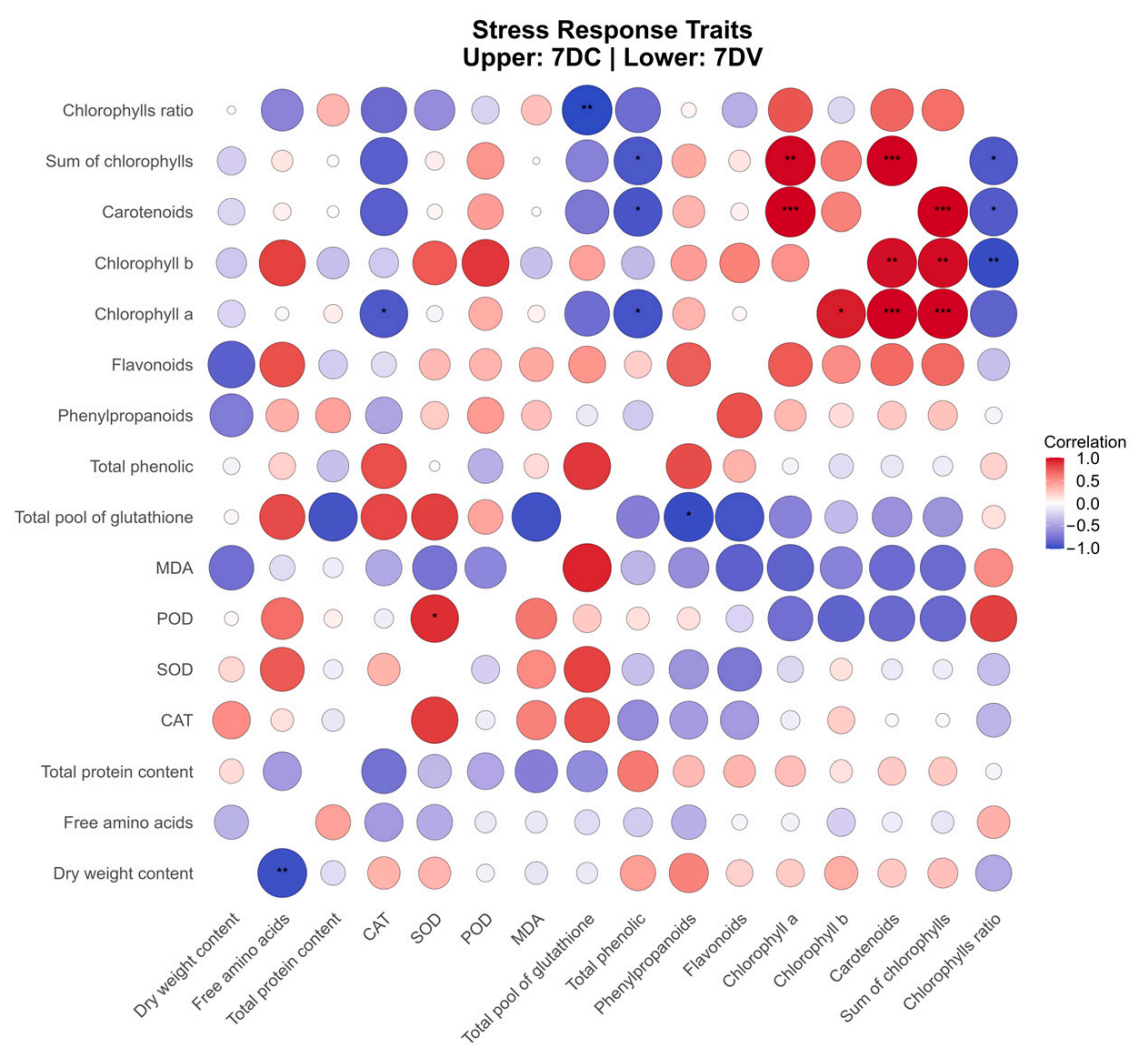
Pearson correlation matrices showing relationships among the biochemical, antioxidant, and phenolic in tobacco plants under control (mock-inoculated, 7DC) and virus-inoculated (7DV) conditions. The upper triangle represents correlations in 7DC, whereas the lower triangle represents correlations in 7DV. Bubble size represents the absolute strength of the Pearson correlation coefficient (|r|), and color indicates correlation direction (blue = negative, red = positive). Significance levels are indicated as: ‘*’, ‘**’ or ‘***’, for *p* < 0.05, *p* < 0.01, and *p* < 0.001, respectively. Correlations were calculated using *n* = 5 biological replicates per treatment; pairwise complete-observations analyses were used where necessary.

Pearson correlation analysis revealed distinct patterns of association among biochemical parameters in mock-inoculated plants and virus-inoculated plants at 7 DPI (7DC and 7DV, respectively) (Figure 5). Overall, virus infection substantially altered the relationships between non-enzymatic antioxidants, free amino acids content, dry weight and photosynthetic pigments. In control plants (7DC; upper triangle), chlorophyll *a* content was negatively correlated with CAT activity and total phenolic content. POD activity was positively correlated with SOD activity, while chlorophyll ratio was negatively correlated with total pool of glutathione. The sum of chlorophyll and carotenoid content was negatively correlated with total phenolic content. The positive correlation was observed between sum of chlorophylls, chlorophyll *a* content and carotenoid content. Moreover, carotenoid content was positively correlated with sum of chlorophylls.

In virus-inoculated plants (7DV; lower triangle), several correlation patterns were markedly altered. Dry weight content showed negative correlation with free amino acids, similar for phenylpropanoids content and total pool of glutathione. Chlorophyll *b*, carotenoid content and sum of chlorophylls positively correlated with chlorophyll *a* content. Same pattern was observed for carotenoid content and the sum of chlorophylls correlated with chlorophyll *b.* Also, the sum of chlorophylls positively correlated with carotenoid content. Contrarily, chlorophyll ratio was negatively correlated with chlorophyll *b* and carotenoid content, as well as with sum of chlorophylls.

Overall, the correlation analysis demonstrated that virus infection reorganized the association network among biochemical and photosynthetic traits.

### 2.6. Expression of Genes Associated with Viral Infection

For the genetic response to viruses in *N. tabacum* and molecular marker selection by RT-PCR, primers were generated for twenty genes from various categories, and a gene encoding a ubiquitin-conjugating enzyme (*Ntubc2*) was chosen as a housekeeping gene (Table 2). All the newly generated primers were verified by RT-PCR analysis, and the melting points of the amplified products were obtained as shown in Table S1.

**Figure 6:**
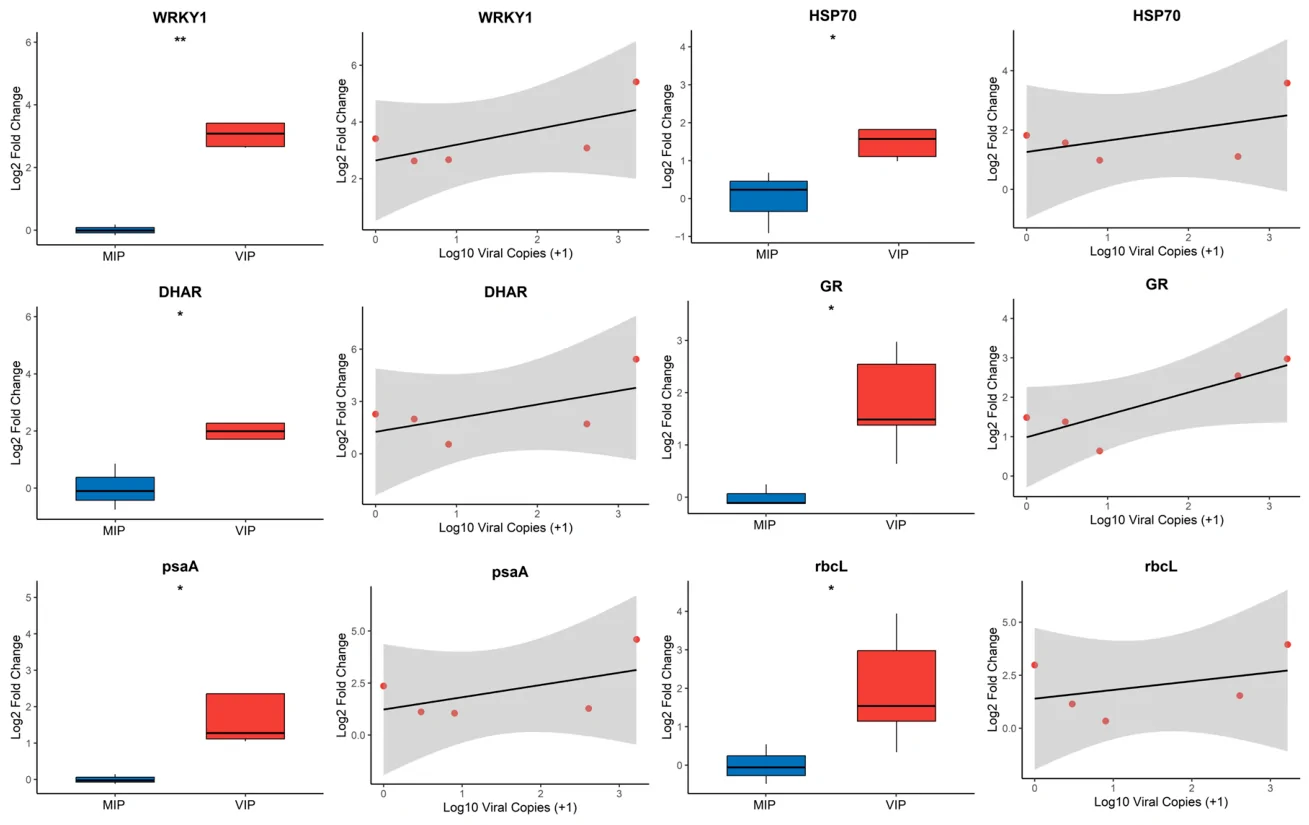
Differential expression of virus-responsive genes and their correlations with viral load in mock-inoculated plants (MIPs) and virus-inoculated plants (VIPs). Gene expression is shown as log_2_ fold change relative to control plants. * *p* < 0.05, ** *p* < 0.01. Correlations between gene expression and log_10_-transformed viral copy number were calculated.

Expression analysis revealed that all six genes (*DHAR*, *GR*, *HSP70*, *psaA*, *rbcL*, and *WRKY1*) responded to virus infection at 7 DPI compared with the non-infected control plants (Figure 6). The boxplots in the left panels of the figure show each gene’s log_2_ fold-change distribution relative to the control. The gene *WRKY1*, as the defense- and pathogenesis-related marker, showed clear upregulation. The stress indicator *HSP70* displayed clear induction. The expression of the antioxidant and redox-defense genes *DHAR* and *GR* increased in both cases. *DHAR* was regulated moderately, and the variability among biological replicates suggests responsive but not uniform induction. *GR* showed a strong upregulation in response to virus infection. The chloroplast-encoded genes *psaA* and *rbcL* were significantly upregulated at 7 DPI compared with the non-infected controls. Both genes also showed a positive correlation with viral copy numbers, indicating that plants with higher viral loads exhibited stronger induction of these photosynthetic transcripts.

The correlations between gene expression and viral load shown in the right panels of Figure 6 depict linear regressions between the log_2_ fold change of each gene and log_10_ viral copy number (+1). These regressions reveal how closely individual gene responses track viral accumulation at 7 DPI. The defense-activated genes *DHAR*, *GR*, *HSP70*, and *WRKY1* all exhibited linear correlation trends with viral copy numbers. The photosynthesis-related genes *psaA* and *rbcL* demonstrated linear correlations, consistent with the induction of photosynthetic capacity in the initial infection period.

## 3. Discussion

The present study provides a multi-layered characterization of *Nicotiana tabacum*’s responses to tomato mosaic virus (ToMV) infection by integrating symptom development, viral accumulation dynamics, oxidative stress responses, antioxidant metabolism, photosynthetic performance, correlation networks, and transcriptional reprogramming. By combining biochemical, physiological, and molecular datasets, the results enable a systems-level interpretation of early compatible plant–virus interactions.

### 3.1. Viral Accumulation and Symptom Onset During ToMV Infection

The asymptomatic phase observed at 7 DPI confirms that ToMV replication precedes visible disease symptoms. This observation is consistent with previous reports showing that tobamovirus replication and systemic movement occur before visible tissue damage becomes apparent [19]. The detection of viral RNA in 80% of plants during this symptomless stage highlights the epidemiological importance of early infection monitoring. As shown in our study, the Ct values at 7 DPI indicate active viral replication despite uneven plant colonization. The stabilization of viral accumulation between 14 and 21 DPI suggests the establishment of a systemic infection equilibrium. The plateau in viral copy number has been associated with host metabolic limitations and the activation of defense pathways restricting further viral spread [20].

A study published by Rabie et al. [4] demonstrated that TMV accumulation in tomato increased progressively between 2 and 14 DPI, with the viral load peaking before maximum symptom severity. Similar observations have been reported for ToBRFV (tomato brown rugose fruit virus, *Tobamovirus fructirugosum*), where increasing viral RNA levels were closely associated with the development of systemic symptoms [21,22]. Our results closely follow this pattern, as viral accumulation increased before the appearance of visible symptoms and plateaued after symptom development. This suggests that the early phase of infection is critical for determining subsequent disease progression, with host defense responses activated during this period likely contributing to the restriction of further viral multiplication and spread [23]. Our observations are also consistent with studies showing that extensive host reprogramming begins before the appearance of visible symptoms. For example, transcriptomic analyses of compatible virus–host interactions have demonstrated that defense-related pathways are activated during early systemic infection, whereas visible symptom development occurs later and reflects the cumulative outcome of these early molecular changes rather than their onset [24].

### 3.2. Redox Imbalance and Antioxidant Regulation During ToMV Infection

The significant increase in malondialdehyde (MDA) at 7 DPI demonstrates that oxidative membrane damage developed during the asymptomatic phase of ToMV infection. This observation is consistent with the role of reactive oxygen species (ROS) as early mediators of antiviral signaling and oxidative stress during compatible plant–virus interactions [25,26]. Studies on PMMoV (pepper mild mottle virus, *Tobamovirus capsici*) have similarly shown that oxidative stress increases with infection severity and is associated with symptom development [27]. In our study, the elevated MDA levels indicate that ROS production had already exceeded the constitutive antioxidant protection capacity at the early stage of infection. However, the persistence of elevated MDA despite enhanced antioxidant activity suggests that oxidative balance was only partially restored under the applied experimental conditions [5,25]. Early induction of oxidative stress has also been reported in other compatible plant–virus interactions, where ROS accumulation precedes symptom development and functions both as a defense signal and as a driver of subsequent physiological disturbances. Similar observations indicate that oxidative imbalance is one of the earliest measurable host responses to viral infection and may contribute to downstream changes in photosynthesis, metabolism, and defense signaling [28].

Enhanced POD and SOD activities indicate the activation of enzymatic antioxidant defenses during early ToMV infection. Superoxide dismutase catalyzes the conversion of superoxide radicals into hydrogen peroxide, whereas peroxidases contribute to hydrogen peroxide detoxification and cell wall reinforcement [29]. In contrast, catalase activity did not increase significantly, suggesting that the antioxidant response was selective rather than globally activated. Such differential regulation may allow ROS to retain their signaling function while limiting excessive oxidative damage [5]. Similar patterns have been reported during viral infections, where antioxidant enzymes not only participate in cellular protection but also influence the outcomes of virus–host interactions [10,30].

The reduction in free amino acids without changes in total protein content suggests metabolic reallocation rather than protein degradation. Viral replication and defense responses both require substantial nitrogen resources; therefore, the maintenance of total protein content may reflect compensatory synthesis of viral and stress-related proteins [13].

Despite oxidative stress induction, glutathione levels remained stable, suggesting efficient recycling within the ascorbate–glutathione cycle. The upregulation of GR and DHAR genes supports this interpretation and indicates transcriptionally controlled redox buffering. The maintenance of glutathione homeostasis is considered a hallmark of successful acclimation to biotic stress [6]. Glutathione is a key component of the cellular redox buffer and participates in the ascorbate–glutathione cycle, linking enzymatic and non-enzymatic antioxidant defenses [5,31]. Its significant associations with both oxidative stress markers and antioxidant enzymes suggest dynamic involvement in maintaining redox homeostasis and possibly in signaling processes regulating stress responses. Moreover, negative correlation between total pool of glutathione and phenylpropanoids content may indicate the importance of balancing and producing non-enzymatic antioxidants under ToMV infection conditions.

Phenolic and phenylpropanoid pools were unchanged, whereas flavonoids decreased significantly. Flavonoids act as ROS scavengers and antiviral metabolites; however, their synthesis requires substantial carbon investment [12]. Reduced flavonoid accumulation may therefore reflect carbon diversion toward defense signaling and viral replication processes. Importantly, the stable levels of total phenolics and phenylpropanoids suggest that ToMV infection did not suppress the phenylpropanoid pathway globally. Rather than reflecting an overall reduction in phenolic metabolism, our results indicate a redistribution of metabolic flux within the phenylpropanoid pathway. Such selective metabolic reprogramming is consistent with the current understanding that plant responses to biotic stress involve the dynamic reallocation of metabolic resources toward specific branches of specialized metabolism, rather than uniform changes across the entire phenolic pool [32]. Recent studies have demonstrated that phenylpropanoid metabolism exhibits remarkable plasticity, allowing individual branches of the pathway to be differentially regulated depending on the nature of the stress and the physiological requirements of the plant [32]. Consequently, the observed decrease in flavonoids despite unchanged total phenolic and phenylpropanoid contents may indicate the preferential allocation of metabolic intermediates toward other phenylpropanoid-derived compounds involved in defense, signaling, or cell wall reinforcement, although targeted metabolomic analyses will be required to verify this hypothesis.

### 3.3. Photosynthetic Dysfunction and Chloroplast-Associated Gene Expression During ToMV Infection

Infection with ToMV induces severe physiological and biochemical disturbances in leaves, including growth inhibition, tissue deformation, oxidative stress, hormonal imbalance, and impaired photosynthesis. It disrupts the entire photosynthetic apparatus, affecting PSII, PSI, and the linear electron transport chain [16,33,34,35]. At 7 DPI, the studied plants showed significant upregulation of *WRKY1*. This gene promotes senescence by activating salicylic acid signaling and inducing senescence-associated genes (SAGs), including SAG12 [36,37]. Accordingly, in our experiment, chlorophyll and carotenoid levels declined, while the MDA content and SOD and POD activities increased. Strong correlations among photosynthetic pigments indicate tightly coordinated metabolism, whereas the relationships between MDA and pigment traits support the role of ROS-induced oxidative stress in destabilizing the photosynthetic apparatus.

ToMV targets the PsbO protein of the oxygen-evolving complex (OEC), causing donor-side limitation [38]. However, our experiment revealed that, seven days after infection, infected leaves showed increased *GR1* and *DHAR* transcription. The encoded enzymes protect the OEC from oxidative damage and maintain redox homeostasis, preventing donor-side limitation of PSII.

The ToMV coat protein localizes to thylakoid membranes, destabilizing PSII reaction centers [16,39,40]. Accordingly, infected tobacco plants showed more unstable PSII reaction centers (RC/CSo) and, despite higher light absorption (ABS/CSo) and energy trapping (TRo/CSo), increased energy dissipation (DIo/CS).

ToMV damages plastoquinone (PQ) and destabilizes the cytochrome b_6_f complex, reducing electron transport between PSII and PSI, which leads to PSI oxidation and acceptor-side limitation of PSII. Consistently, infected leaves showed decreased electron transport efficiency beyond PSII (ETo/CS; ETo/RC). ToMV also impairs Rubisco by inhibiting RbcS transport and downregulating *RbcS* and *RbcL* expression [41,42]. In our experiment, a significant increase in *RbcL* transcription was observed seven days post-infection, indicating that defense responses remained at least partially effective.

Defense- and redox-related genes (*DHAR*, *GR*, *HSP70*, *WRKY1*) increased proportionally with ToMV accumulation. Photosynthesis-related genes (*psa*A, *rbc*L) were also upregulated, with *rbc*L showing the strongest response, suggesting enhanced carbon assimilation. These results indicate that viral accumulation is closely associated with the activation of antioxidative defense pathways and transient chloroplast reprogramming. Although chloroplast genes are typically downregulated during viral infection, our experiment suggested that early infection stages may induce compensatory or virus-driven upregulation. Accordingly, at 7 DPI, *psa*A and *rbc*L transcription was transiently activated, reflecting increased chloroplast activity and temporary stimulation of photosynthesis before the later decline associated with chlorosis [43].

The apparent discrepancy between increased expression of chloroplast-associated genes and reduced photosynthetic performance observed in our study agrees with previous reports indicating that transcriptional responses during the early stages of compatible virus infection do not necessarily translate into immediate functional recovery. Instead, transient activation of chloroplast-related genes appears to represent an early compensatory response that precedes the progressive decline in photosynthetic efficiency accompanying symptom development [28].

### 3.4. Study Limitations and Future Perspectives

This study has provided new insights into early ToMV infection by documenting asymptomatic viral replication, oxidative stress responses, and changes in chloroplast-related gene expression, with particular emphasis on the early infection stage (7 DPI). The targeted analysis of selected antioxidant enzymes, stress markers, and key genes enabled the identification of initial host responses and suggested the activation of specific defense- and photosynthesis-related pathways. However, the experimental design was limited to discrete timepoints, which constrains a full understanding of the dynamic progression from early compensation to irreversible damage. A limitation of the present study is that transcriptomic analyses were conducted at a single timepoint (7 DPI). Although this stage represents an early phase of pathogen colonization, as indicated by the continuing increase in viral load and the absence of detectable virus in some inoculated plants, the lack of a time-course analysis prevents the assessment of the temporal dynamics of host responses and does not capture the full range of molecular events occurring during the initial stages of infection.

Future work will expand upon these findings by increasing the temporal resolution, particularly at very early (1–3 DPI) and later infection stages, to better define the transition phases of disease development. A broader systems-level approach will be implemented, integrating transcriptomic, proteomic, and metabolomic analyses to uncover additional regulatory networks and to distinguish host defense mechanisms from virus-induced metabolic reprogramming. Further studies will focus on elucidating the mechanistic links between oxidative stress and chloroplast function, including the observed transient upregulation of photosynthesis-related genes despite structural damage. Clarifying whether this response reflects host compensation or viral manipulation will be critical. Additionally, future research will explore the dual role of reactive oxygen species and antioxidant systems, with the aim of identifying targets for improving plant resistance, such as redox regulation pathways, antioxidant capacity, and key transcription factors including WRKY proteins.

## 4. Materials and Methods

### 4.1. Plant Cultivation, Inoculation, and Sample Collection

Tobacco plant (*Nicotiana tabacum* ‘Samsun’, fully sensitive to infection with ToMV—nn genotype) seeds were sown in a medium containing a mixture of soil and sant (3:1, *v*:*v*) in containers. After 10 days, seedlings were transferred to pots with a diameter of 12 cm with soil and sand (3:1, *v*:*v*) and placed in a growing room with a controlled environment. The environmental conditions were as follows: a 16/8 photoperiod (day/night), a temperature of 22 ± 1 °C, and white fluorescence light characterized by a photosynthetic photon flux density (PPFD) of 120 µmol × m^−2^ × s^−1^. Plants at the four-leaf stage were inoculated with ToMV. The ToMV used for inoculation was isolated from *Physalis* sp. plants grown in Krakow (Poland) [44]. Since its isolation, the virus has been replicated annually in tobacco plants, where it exhibited its classic symptoms. Plant material infected with the virus at full systemic symptoms (21 DPI) was collected and stored at −20 °C.

**Figure 7:**
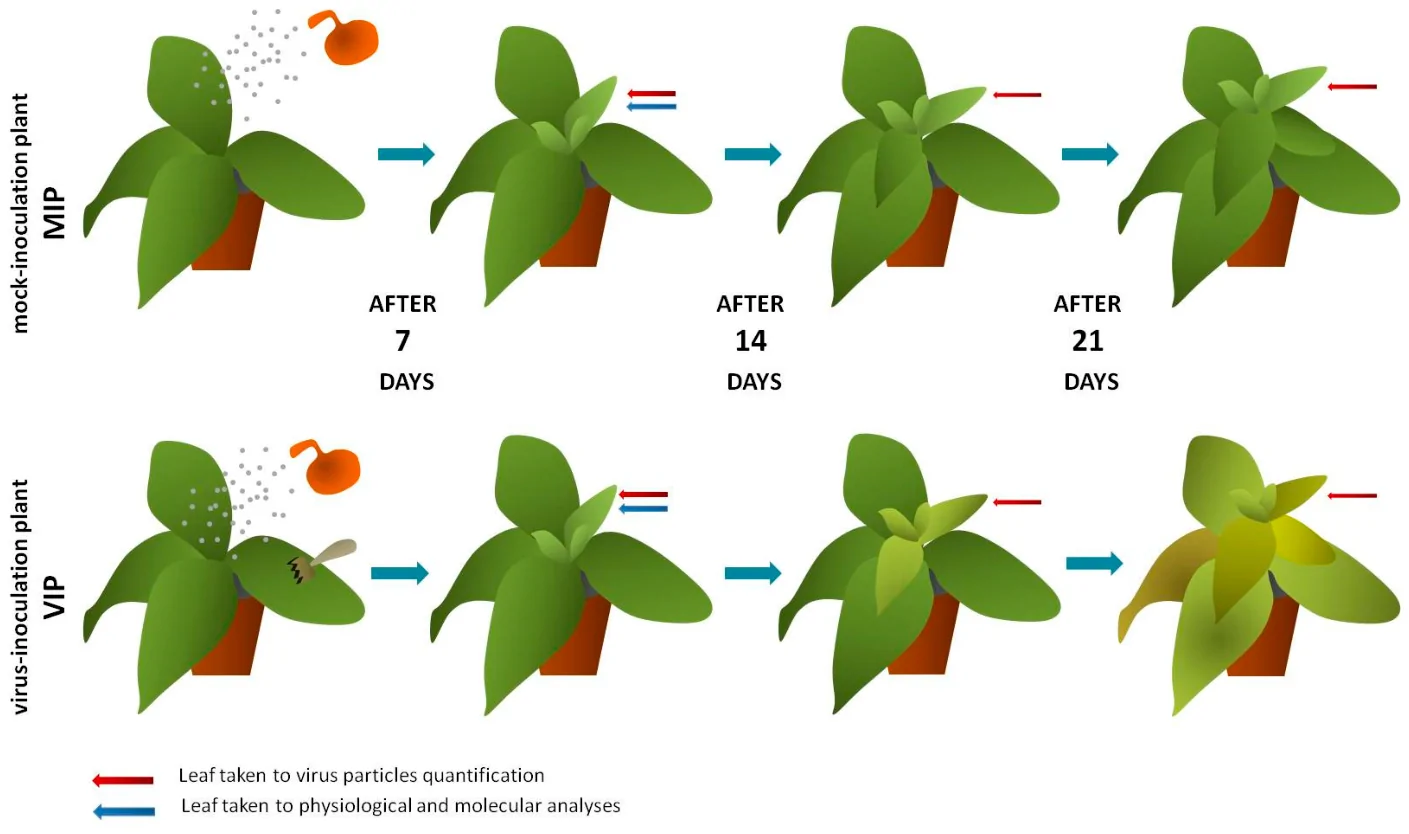
Schematic diagram of the research conducted.

ToMV inoculum was prepared from virus-infected leaf material, which was ground in inoculation buffer (0.1 M sodium phosphate, 2% (*w*/*v*) PVP, 0.2% (*w*/*v*) Na_2_SO_3_) at a ratio of 1:10. The inoculum was divided into 2 mL portions in thin-walled glass tubes and heated at 83 °C for 15 min as a selective treatment exploiting the exceptional thermal stability of ToMV, thereby reducing the likelihood of contamination by less heat-stable viruses. This prepared inoculum was then used to infect plants. Leaves were dusted with carborundum (particle size, 0.037 mm). A 1 mL volume of viral extract or buffer (mock-inoculated control) was applied by gently rubbing the upper surface of the leaves (Figure 7). Each group (mock- and virus-inoculated) contained 20 plants (*n* = 20).

To confirm successful ToMV infection and increasing of virus particles, individual inoculated plants were screened at 7, 14 and 21 days post inoculation (DPI) prior to inclusion in subsequent analyses (Section 4.2).

At 7 DPI, the youngest fully developed leaf that developed over the inoculated leaves was used for the chlorophyll *a* fluorescence measurement and then harvested for further biochemical and molecular analysis. Because infection was confirmed in 80% of plants after 7 DPI (4 plants with negative and 16 plants with positive RT-PCR), and 1 of the infected plants was used for detailed photographic documentation, 15 infected plants were used for further analysis. The same number of control plants were included for further analysis. The youngest, fully developed leaf was collected from each plant within each group. The leaves were then combined in groups of three, resulting in five independent biological samples (*n* = 5). The same was performed to collect samples 14 and 21 days after inoculation for RNA isolation and virus load quantification. Pooled samples were immediately frozen in liquid nitrogen, homogenate and stored at −80°C. Part of the tissues for biochemical analysis were then freeze-dried for 24 h. The analytical samples for the biochemical analyses were prepared from the plant material thus prepared, where each biochemical analysis consisted of 5 independent biological repetitions (*n* = 5).

### 4.2. Virus Detection in Inoculated Plants by RT-PCR with Absolute Quantification

To confirm successful Tomato mosaic virus (ToMV) infection and increasing of virus particles, individual inoculated plants were screened at 7, 14 and 21 days post inoculation (DPI) prior to inclusion in subsequent analyses.

Plants producing the expected ToMV-specific amplification signal were classified as infected and were included in subsequent biochemical and gene expression analyses. Plants testing negative for ToMV were excluded from further experiments. Consequently, all pooled biological replicates consisted exclusively of plants with confirmed viral infection.

#### 4.2.1. Quantitative RT-PCR

The youngest fully developed leaves that had emerged above the inoculated leaves were sampled for total RNA extraction. Total RNA was extracted using the GeneJET Plant RNA Purification Mini Kit (Thermo Scientific, Vilnius, Lithuania) according to the manufacturer’s instructions. RNA quantity and purity were assessed using NanoDrop spectrophotometry and Qubit fluorometry with the Qubit dsRNA HS Assay Kit (Invitrogen, Eugene, OR, USA). The isolated RNA was used for first-strand cDNA synthesis with the Maxima H Minus First Strand cDNA Synthesis Kit (Thermo Fisher Scientific, Waltham, MA, USA).

ToMV presence was determined by quantitative real-time PCR (qRT-PCR) using ToMV-specific primers amplifying a 298 bp fragment of the coat protein gene (5′-CACTTCTCCATCGCAATTTGTGT-3′ and 5′-TGTCGGATTCTGCTGGTTTTCTA-3′) and SYBR Green Master Mix with standard thermocycling mode (60 °C for annealing and extension). Amplification reactions were performed in a final volume of 10 μL containing 5 μL of SYBR Green Master Mix, 0.3 μM of each primer, 1 μL of diluted cDNA template, and nuclease-free water. Reactions were performed in Applied Biosystems QuantStudio 5 Real-Time PCR System (Thermo Fisher Scientific, Waltham, MA, USA). Melting curve analysis was performed at the end of the run to verify amplicon specificity, with a characteristic melting temperature (Tm) of 81.0 °C.

#### 4.2.2. Construction of External Calibration Curve

A quantitative PCR (qPCR) calibration curve was generated using a plasmid construct containing the target virus coat protein (CP) gene sequence. The plasmid DNA concentration was quantified fluorometrically using a double-stranded DNA specific detection assay—Qubit 4.0 with the 1xdsRNA HS Assay Kit (Invitrogen, Eugene, OR, USA). The absolute plasmid copy number was calculated based on its molecular weight and Avogadro’s constant. The stock plasmid preparation was serially diluted in seven-fold dilution steps to obtain a standard range spanning from 10 to 10^7^ copies per µL. Each dilution point was run in technical triplicates using the same qPCR reaction mix and cycling conditions as applied to the experimental samples. For each dilution point, the threshold cycle (Ct) value was determined. A linear regression was generated by plotting Ct against log_10_ (copy number). The regression line was calculated using the least squares method, producing the calibration curve equation:

y = −3.6614x + 29.923

where y = Ct value; x = log_10_ (copy number).

The resulting coefficient of determination (R^2^ = 0.9986) indicated excellent linearity and a highly reliable Ct and copy number relationship.

PCR amplification efficiency (*E*) was calculated using the standard formula:

$$E=(10^{-1/slope}-\;1)\;\times \;100\%$$

The obtained amplification efficiency was 88%, which is acceptable for viral quantification assays in agronomy. For all qPCR reactions, 1 µL of extracted nucleic acid was used as the template with the PowerUp™ SYBR™ Green Master Mix (Thermo Fisher Scientific, Waltham, MA, USA) with standard thermocycling mode (60 °C for annealing and extension). Applied Biosystems QuantStudio 5 (Thermo Fisher Scientific, Waltham, MA, USA) was used for thermocycling. Accordingly, the calculated copy numbers directly represent copies per µL of the original sample extract.

### 4.3. Biochemical Analysis in Virus-Infected Plants

#### 4.3.1. Malondialdehyde Content Analysis

The lipid peroxidation level was estimated using the malondialdehyde (MDA) content method [45], with the modifications of Blat et al. [46]. A 15 mg amount of DW plant material was extracted with 1 mL of 0.1% trichloroacetic acid (TCA) at 4 °C and centrifuged for 15 min (25,155× *g*, 4 °C). Reaction mixtures (0.2 mL of extract and 0.8 mL of 0.5% thiobarbituric acid (TBA) in a 20% TCA solution) were incubated for 30 min at 95 °C, cooled on ice, and centrifuged for 10 min (25,155× *g*, 4 °C). The absorbance of the samples was measured at 532 nm and 600 nm using a Unico 2804 UV spectrophotometer. The MDA content was calculated according to Dhindsa et al. [45] using the MDA extinction coefficient (ε = 155 mM·cm^−1^). The absorbance for the reaction mixture at 532 nm was reduced by the correction value obtained at 600 nm. The results are expressed as nmol of MDA per 1 g of DW. Analyses were performed in five replicates.

#### 4.3.2. Free Amino Acids Content Analysis

The content of free amino acids in the tobacco tissue was determined according to Kamińska et al. [47], with some modifications. Briefly, 0.2 mL of the methanolic extract was mixed with 0.5 mL of 0.2% ninhydrin in isopropanol and 0.3 mL of isopropanol. Test tubes with the mixtures were vortexed and incubated at 85 °C for 15 min. The absorbance was recorded at 570 nm. Glycine was used for the standard curve. The results are expressed as micrograms of glycine per 1 g of DW. Analyses were performed in five replicates.

#### 4.3.3. Total Protein Content and Enzymatic Antioxidant Activity Analysis

To estimate the protein content and activity of CAT, POD, and SOD, native proteins were extracted from dry plant material (20 mg), using 2 mL of potassium phosphate buffer (0.05 M, pH = 7.00) with 10 μL of 1 M DTT, 11 mg of EDTA, 0.2 g of PVPP, 100 μL of Triton X-100, and 1 mM PMSF (0.0174 g·100 mL) dissolved in a few drops of alcohol per 10 mL of buffer, at 4 °C. The samples were centrifuged for 15 min at 25,155× *g*, 4 °C. The protein concentration was assessed according to Bradford [48], with modifications by Makowski et al. [49]. A calibration curve was prepared using bovine serum albumin (BSA) as the standard. The SOD activity was measured spectrophotometrically according to Hwang et al. [50], with Wiszniewska et al.’s [51] modifications. The reaction mixtures were prepared by mixing 2.18 mL of 100 mM phosphate buffer, pH 7.8; 0.2 mL of 55 mM methionine; 0.4 mL of 0.75 mM nitrotetrazole blue (NBT); 0.2 mL of 0.1 mM riboflavin; and 20 μL of protein extract. The reaction was started by placing the samples under a 40 W lamp at room temperature. The absorbance of the mixtures was measured 5 and 10 min after starting the reaction at 560 nm. Samples without extract were the control, where the activity was 100%. Activity is expressed as U per 1 g of DW, where U means the inhibition of the reaction by 50% in relation to the control. The POD activity was estimated using the spectrophotometric method of Lűck [52], with the modifications of Tokarz et al. [53]. A total of 1 mL of diluted extract was mixed with sodium phosphate buffer (1 mL), pH 6.2; 0.1 mL of pPD; and 0.2 mL of H_2_O_2_. The absorbance of the samples was measured at 485 nm. The POD activity is expressed as U per minute per 1 g of DW, where 1U means a 0.1 increase in absorbance. To estimate CAT activity, tissue was extracted in 1 mL of phosphate buffer (pH 7.0) and centrifuged for 10 min (25,155× *g*, 4 °C). The reaction mixture consisted of 0.2 mL of the extract, 1.8 mL of phosphate buffer, and 1 mL of 0.3% H_2_O_2_ solution in phosphate buffer. The absorbance of the mixture was measured for 4 min at 1 min intervals at 240 nm [54]. Catalase activity is expressed in units as the amount of enzyme that decomposes 1 μmol of H_2_O_2_ in 1 min. All the described analyses were performed in five repetitions.

#### 4.3.4. Glutathione Pool Estimation

The total glutathione pool (reduced glutathione (GSH) + glutathione disulfide (GSSG)) was analyzed according to Queval and Noctor [55], with the modifications of Makowski et al. [49]. A 10 mg amount of dry tissue was extracted in 1 mL of 0.5 M HCl at 4 °C and centrifuged for 15 min (25,155× *g*, 4 °C). The pH of 400 μL of the obtained extract was neutralized with 0.2 M NaOH or 0.5 M HCl until it reached a value between 5.5 and 6.0. Then, 30 μL of the neutralized extract, 300 μL of buffer with EDTA (pH 7.5), 30 μL of 10 mM NADPH, 30 μL of 12 mM DTNB, and 180 μL of MiliQ water were mixed. To each reaction mixture 30 μL of glutathione reductase (GR) (20 U·μL^−1^) was added. The absorbance of the mixtures was measured at 412 nm, 1 min and 2 min after the addition of GR. The results are expressed as mmol of GSH + GSSG per 1 g of DW. Analyses were repeated in five repetitions.

#### 4.3.5. Total Phenolic, Phenylpropanoid, and Flavonoid Contents

The total phenolic content was assessed using Folin–Ciocalteu reagent [56], with modifications according to Miernicka et al. [57]. The phenylpropanoid and flavonoid accumulation was estimated using the method of Fukumoto and Mazza [58], with modifications [59]. A 20 mg amount of freeze-dried plant material was extracted in 2 mL of 80% methanol by sonication in an ultrasonic bath (POLSONIC 2, Warsaw, Poland) for 30 min. The samples were centrifuged for 15 min (25,155× *g*, 4 °C). To estimate the total phenolic content, 1 mL of 25-fold-diluted extract was mixed with 0.2 mL of Folin’s reagent (Sigma-Aldrich Chemie, GmBH, Steinheim, Germany) and 1.6 mL of 5% Na_2_CO_3_, and incubated for 20 min at 40 °C. The absorbance of the samples was measured at 740 nm. Chlorogenic acid (Sigma-Aldrich Chemie, GmBH, Steinheim, Germany) was used as a reference standard. The results are expressed as milligrams of chlorogenic acid equivalents per 1 g of DW. For phenylpropanoid and flavonoid estimation, plant tissue was extracted as in the method for total phenolic content estimation. The supernatant was mixed with 0.25 mL of 0.1% HCl in 96% EtOH and 4.55 mL of 2% HCl in H_2_O. Samples were incubated at room temperature (darkness) for 20 min. The absorbance was measured at wavelengths of 320 and 360 nm. The contents of phenylpropanoids and flavonoids were calculated using calibration curves made for caffeic acid and quercetin (Sigma-Aldrich Chemie, GmBH, Steinheim, Germany), respectively. The results are expressed as milligrams of standard equivalents per 1 g of DW. All analyses were performed in 5 replicates.

#### 4.3.6. Photosynthetic Pigment Content Estimation

Photosynthetic pigments were extracted and measured according to the Lichtethaler [60] method, with the modifications of Makowski et al. [61]. Approximately 15 mg of freeze-dried leaf tissue was homogenized with 1 mL of 80% acetone at 4 °C. The samples were centrifuged (25,155× *g*, 4 °C). Then, the supernatant was collected and the pellet was extracted with 0.5 mL of 80% acetone twice. In total, 2 mL of extract was collected and diluted ten-fold with 80% acetone. The absorbance of the extract was measured at 663, 646, and 470 nm. The contents of Chl *a*, Chl *b*, and Car were calculated according to the Wellburn [62] equations. The content of photosynthetic pigments is expressed as mg per g of DW. The analyses were repeated five times.

#### 4.3.7. Chl a Fluorescence

The chl *a* fluorescence was measured on the leaves using a Handy PEA spectrofluorometer (Hansatech Instruments, King’s Lynn, UK) according to standard procedures. The leaves were dark-adapted for approximately 20 min and then exposed to a red light of a wavelength λ_max_ = 650 nm and an intensity of 2000 μmol of photons∙m^−2^∙s^−1^. Selected functional and structural photosynthetic parameters were calculated [63,64,65]. The calculated parameters were the specific (per reaction center, RC) and phenomenological (per excited cross-section, CS) energy fluxes for absorption, trapping, and electron transport:

ABS/RC: Absorption flux per RC corresponding directly to its apparent antenna size.
TRo/RC: Trapping flux leading to QA reduction per RC at t = 0.
ETo/RC: Electron transport flux from QA^−^ to plastoquinone per RC at t = 0.
DIo/RC: Dissipated energy flux per RC at the initial moment of the measurement, i.e., at t = 0.
RC/CSo: Density of active PS II reaction centers per cross-section approximated by Fo.
RC/CSM: Density of active PS II reaction centers per cross-section approximated by FM.
ABS/CSo: Absorption of energy per excited cross-section (CS) approximated by Fo.
TRo/CSo: Excitation energy flux trapped by PSII of a photosynthesizing sample cross-section (CS) approximated by Fo.
ETo/CSo: Electron flux transported by PSII of a photosynthesizing sample cross-section (CS) approximated by Fo.
DIo/CSo: Heat dissipation of excitation energy by PSII of a photosynthesizing sample cross-section (CS) approximated by Fo.
ABS/CSm: Absorption of energy per excited cross-section (CS) approximated by FM.
TRo/CSm: Excitation energy flux trapped by PSII of a photosynthesizing sample cross-section (CS) approximated by FM.
ETo/CSm: Electron flux transported by PSII of a photosynthesizing sample cross-section (CS) approximated by FM.
DIo/CSm: Heat dissipation of excitation energy by PSII of a photosynthesizing sample cross-section (CS) approximated by FM.

### 4.4. Gene Expression Analysis in Virus-Infected Plants

#### 4.4.1. RNA Sampling and cDNA Synthesis

Five individual biological samples (*n* = 5) from the treatment groups—control (MIPs) and with virus (VIPs) 7 DPI—were taken to ensure an adequate number of biological replicates. The sampling procedure was as described above in Section 4.1. To control potential aging or time-dependent effects unrelated to virus infection, plant material was sampled from an uninfected control group at the same timepoint as the treatment. The sampled plant material was frozen in liquid nitrogen and stored at −80 °C for RNA extraction. Total RNAs were isolated by using a GeneJET Plant RNA Purification Mini Kit (Thermo Scientific, Vilnius, Lithuania) following the manufacturer’s instructions. The quantity and purity of the RNA were assessed using NanoDrop spectrophotometry and Qubit fluorometry with the 1xdsRNA HS Assay Kit (Invitrogen, Eugene, OR, USA). The isolated RNA was employed to synthetize first-strand cDNA using the Maxima H Minus First Strand cDNA Synthesis Kit (Thermo Fisher Scientific, Waltham, MA, USA).

#### 4.4.2. RT-PCR Analysis of Infected Plants

The quantitative real-time PCR (RT-PCR) on five biological (*n* = 5) and three laboratory replicates was performed using the PowerUp™ SYBR™ Green Master Mix (Thermo Fisher Scientific, Waltham, MA, USA) with standard thermocycling mode (60° C for annealing and extension) using Applied Biosystems QuantStudio 5 (Thermo Fisher Scientific, Waltham, MA, USA). The sampling procedure was as described above in Section 4.1. Specific oligonucleotide primers for 21 genes that encode proteins related to biotic stress were designed with Primer3web v4.1.0. The sequences were selected according to analogous genes of *Nicotiana* spp. obtained from the NCBI database (Table S1).

### 4.5. Statistic Analysis and Data Visualization

The experiment was performed twice on fifteen plants per treatment (mock-inoculated (control) and virus-inoculated). All the spectrophotometric estimations were made in five replications. Chl a fluorescence measurement was performed in twenty replications. Significant differences between means were determined using Student’s t-test at *p* < 0.05. STATISTICA 13.3 (StatSoft Inc., Tulsa, OK, USA) was used for the statistical analyses.

Gene expression was analyzed in R (version 4.3.2) using RStudio. The raw Ct values were imported from Excel using the readxl package [66]. Data preprocessing, transformation, and reshaping were carried out using the tidyverse suite (dplyr, tidyr, and stringr) [67]. Relative gene expression was determined using the comparative Ct (ΔCt) method, calculating ΔCt as Ct_target minus Ct_reference (*Ntubc2*). For each gene, the control group’s mean ΔCt served as a calibrator to compute ΔΔCt. Log2 fold changes were derived as −ΔΔCt, with fold changes calculated as 2^−ΔΔCt^. These steps were executed with grouped summaries and mutation functions within dplyr. Statistical analyses used base R functions from the stats package. The differences between groups were tested with one-way ANOVA. The relationships between the gene expression and viral load (log10 of the viral copy number) were examined using Spearman’s correlation and linear regression models. The results were summarized with broom [68], the figures were created with ggplot2 [69], and a statistical table was exported via openxlsx [70].

The correlations between the stress response parameters in tobacco plants under the control (7DC) and virus-inoculated (7DV) conditions were analyzed using R (version 4.3.2 in RStudio). The raw trait data were imported from Excel via readxl [66] and processed with the tidyverse packages (dplyr, tidyr, stringr) to convert the data to numeric format, filter it, and restructure it [67]. Pearson correlation matrices were generated separately for the 7DC and 7DV plants using Hmisc::rcorr [71], with the upper and lower triangles merged into a single matrix for visualization. These correlations were depicted as bubble plots using ggplot2 [69], where the size of a bubble represents the absolute value of the correlation coefficient, the color indicates whether the correlation is positive or negative, and the significance levels are denoted by asterisks (*** *p* < 0.001, ** *p* < 0.01, * *p* < 0.05). For each pairwise correlation, only complete observations were included in the analysis; consequently, the number of observations could vary across variable pairs due to missing values. Pearson correlation coefficients (r), two-sided *p*-values, and 95% confidence intervals were calculated for each pairwise association. The observed differences in correlation patterns between 7DC and 7DV plants were interpreted as differences in the strength and direction of associations among traits and were not considered evidence of causal relationships.

### 4.6. AI-Assisted Writting

The authors used ChatGPT (GPT-5.6 Sol, OpenAI) to assist in drafting the body of the manuscript. AI was used to search for the scientific literature needed to write the manuscript and to edit the Introduction, Discussion, and Abstract sections. The generated text was critically reviewed and edited by the authors to ensure scientific accuracy and compliance with journal standards.

## Figures and Tables

**Table 1:** Contents of non-enzymatic antioxidants: glutathione [mmol/g DW], total phenolics, phenylpropanoids, and flavonoids [mg/g DW] in mock-inoculated plants (MIPs) and virus-inoculated plants (VIPs). The asterisk indicates statistical significance of means acc. Student’s *t*-Test at *p* < 0.05; SD—standard deviation; DW—dry weight.

Non-Enzymatic Antioxidants	MIP	VIP
Total pool of glutathione, mmol/g DW [±SD]	1.39 ± 0.12	1.17 ± 0.15
Total phenolic content, mg/g DW [±SD]	19.21 ± 1.42	18.97 ± 0.79
Phenylpropanoids, mg/g DW [±SD]	8.16 ± 0.48	8.38 ± 0.35
Flavonoids, mg/g DW [±SD]	5.57 ± 0.56 *	4.50 ± 0.20

**Table 2:** Selected panel of genes for ToMV response analysis in 7 DPI *N. tabacum* leaves.

Function Groups	Gene *
Defense- and Pathogenesis-Related Markers	Pathogenesis-Related Protein 1 (*PR1*), WRKY Transcription Factor 70 (*WRKY70*)
Antioxidant and Redox-Defense Genes	Ascorbate Peroxidase 1 (*APX1*), Catalase 1 (*CAT1*), Superoxide Dismutase (*SOD1*), Glutathione Peroxidase (*GPX4*), **Glutathione Reductase** (***GR1***), **Dehydroascorbate Reductase (DHAR)**
Lipid-Derived Signaling/Oxylipin Pathway	Lipoxygenase 1 (*LOX1*)
Non-Enzymatic Antioxidants (Proline, Glutathione and Ascorbate Biosynthesis) Genes	Δ^1^-Pyrroline-5-Carboxylate Synthetase (key enzyme in proline biosynthesis) (*P5CS*), GDP-L-Galactose Phosphorylase (also known as VTC2/VTC5; key enzyme in ascorbate biosynthesis) (*GGP*), Glutamate–Cysteine Ligase (enzyme catalyzing the first step of glutathione biosynthesis) (*GSH1*)
Photosynthesis-Related Genes	**Ribulose-1,5-Bisphosphate Carboxylase/Oxygenase Large Subunit (RuBisCO large subunit)** (***rbcL***), Ribulose-1,5-Bisphosphate Carboxylase/Oxygenase Small Subunit (RuBisCO small subunit) (*rbcS*), Light-Harvesting Complex II Chlorophyll a/b Binding Protein (*Lhcb*), **Photosystem II Protein D1** (***psaA***)
Transcription Factors	**WRKY Transcription Factor 1** (***WRKY1***), NAC Domain-Containing Transcription Factor (NAM/ATAF/CUC family) (*NAC*)
Stress Indicator Heat-Shock and Protein Quality Control	**Heat Shock Protein 70** (***HSP70***)
Reference Gene (Normalization)	*Nicotiana tabacum* Ubiquitin-Conjugating Enzyme 2 (*Ntubc*2)

* Genes showing significant expression are marked in **bold**.

## Data Availability

The data presented in this study are available on request from the corresponding author.

## Supplementary Materials

The following supporting information can be downloaded at https://www.mdpi.com/article/10.3390/ijms27177520/s1.

## Abbreviations

Car	Carotenoids
CAT	Catalase
Chl	Chlorophyll
Ct	Threshold cycle
DHAR	Dehydroascorbate reductase
DIo	Dissipated energy flux
DPI	Days post inoculation
DW	Dry weight
ETo	Electron transport flux
Fo	Minimum fluorescence
FM	Maximum fluorescence
GR	Glutathione reductase
GSH	Reduced glutathione
GSSG	Oxidized glutathione
HSP70	Heat shock protein 70
JA	Jasmonic acid
MDA	Malondialdehyde
MIP	Mock-inoculated plants
nn	Genotype without immunity genes for ToMV
POD	Peroxidase
PPFD	Photosynthetic photon flux density
PSI	Photosystem I
PSII	Photosystem II
RC	Reaction center
RNA	Ribonucleic acid
ROS	Reactive oxygen species
RT-PCR	Reverse transcription polymerase chain reaction
RU	Relative units
RuBisCO	Ribulose-1,5-bisphosphate carboxylase/oxygenase
SA	Salicylic acid
SD	Standard deviation
SE	Standard error
SOD	Superoxide dismutase
TMV	Tobacco mosaic virus
ToBRFV	Tomato brown rugose fruit virus
ToMV	Tomato mosaic virus
TRo	Trapping flux
VIP	Virus-inoculated plants
WRKY	WRKY transcription factor
