# Supplementary material for: Infection with Tomato Mosaic Virus in Nicotiana tabacum cv. Samsun—Physiological and Molecular Approach During Early Stage of Infection
Source: Int J Mol Sci. 2026 Aug 22;27(17):7520. doi: 10.3390/ijms27177520 (PMC13565832; doi:10.3390/ijms27177520)
Supplement: Supplementary file 1 [file ijms-27-07520-s001.zip › ijms-4445522-supplementary.pdf]

Table S1. Primers used for gene expression analysis, including forward and reverse sequences, amplicon size, and reference gene IDs.

Gene	Forward direction 5'- 3'	Revers direction 5'- 3'	Amplikon size, bp	Reference gene ID in NCBI
PR1	PR1_F GTCGAGATGTGGGTCAATGAGA	PR1_R CATTGTTGCACTGAACCCTAGC	142	NM_001425754.1
WRKY70	WRKY70_F TATGTGACCCGAGAAGCTTGTC	WRKY70_R GACTGTGTTTGACTTGTTCGCA	144	KM510344.1
APX1	APX_F GCAAGGACATGGAGCAAACAAT	APX_R GTGAAAGGGAACATCAGGTCCT	154	AF443182.1
CAT1	CAT1_F GACACATGGAAGGGTATGGTGT	CAT1_R TAGTTTCCAGCAGCAATCGAGT	193	XM_016653320.2
SOD1	SOD1_F CCACAATCCATCATTGGAAGAGC	SOD1_R CCGATGATACCACAAGCAAC	125	XM_016594235.2
GPX4	GPX4_F AGGTTCCAGGATTAAGTGGAAT	GPX4_R ATCGTAAGTTTCATAGACTGCAC	178	XM_075244157.1
GR1	GR_F ATGAAATGCGAGCTGTAGTTGC	GR_R CCTTCCATCAAAGCAACTGGTG	325	XM_016597121.2
DHAR1	DHAR_F GTTGCTGTCGGTTCCAAAAGAA	DHAR_R CAATGTCAGAAGTGCCCTTTGG	183	NM_001326056.1
P5CS	P5CS_F CCGTACAAAACCCAAAATCCCC	P5CS_R AGCTCCTGAATCTGCTCACAAA	277	NM_001326050.1
GGP	GGP_F CCATAAACCACCTCCACTTCCA	GGP_R TACTTGTTCTGAAGGGAGACGC	232	XM_016594034.2
GSH1	GSH_F CCAAGGGCTGTGGTTATGTTTG	GSH_R CTGAATCTTCTTGGTCCCAGCT	327	NM_001326285.1
rbcL	rbcL_F AGGAGGTATTCACGTTTGGCAT	rbcL_R AAACGTCCACTGCTGCAAAATT	287	BK010737.1
rbcS	rbcS_F TACCTTCCTGATTTGAGCGAGG	rbcS_R ACCTGGTGACTTGTTGTTCTCA	132	XM_075235239.1
Lhcb	Lhcb_F AGAACGGACGATTGGCTATGTT	Lhcb_R CAAAGTTAGTGGCAAAAGCCCA	141	XM_009620670.4
psaA	psaA-F GCCAATAACCACGTCCACTG	psaA _R GTCAGATGTTTGGGGCAGTG	239	NC_001879.2
WRKY1	WRKY1_F GATCATGGGCCAGTTTCAGC	WRKY1-R CCTCTACGGTCTTTCCCTCC	203	NM_001325583.1
NAC	NAC_F CTTGAGGCTTGACGATTGGG	NAC_R TCATCGACTCGGACGTTTCA	245	HQ413134.1
HSP70	HSP70_F TTTCAGGAAGTGTATGGAGCCC	HSP70_R TCATCACTCCTCCAGCAGTTTC	305	AY372071.1
Ntubc2	Ntubc2_F CTGGACAGCAGACTGACATC	Ntubc2_R CAGGATAATTTGCTGTAACAGATTA	56	ttps://doi.org/10.1093/pcp/pcw171
